# Influence of major trauma and lower limb loss on radiographic progression and incidence of knee osteoarthritis and pain: a comparative and predictive analysis from the ADVANCE study

**DOI:** 10.1186/s13075-026-03739-4

**Published:** 2026-01-26

**Authors:** Oliver O’Sullivan, Fraje Watson, Anthony M J Bull, Susie Schofield, Emma C Coady, Christopher J Boos, Paul Cullinan, Nicola T Fear, Stefan Kluzek, Alexander N. Bennett, Ana M. Valdes

**Affiliations:** 1 Academic Department of Military Rehabilitation (ADMR), Defence Medical Rehabilitation Centre (DMRC), Stanford Hall, Loughborough, UK; 2 Academic Unit of Injury, Recovery and Inflammation Sciences, Faculty of Medicine and Health Sciences, University of Nottingham, Nottingham, UK; 3 Department of Bioengineering, Imperial College London, London, UK; 4 Centre for Blast Injury Studies, Department of Bioengineering, Imperial College London, London, UK; 5 National Heart and Lung Institute, Imperial College London, London, UK; 6 Faculty of Health & Social Sciences, Bournemouth University, Bournemouth, UK; 7 Academic Department of Military Mental Health, King’s College London, London, UK; 8 Centre for Sport, Exercise and Osteoarthritis Research Versus Arthritis, University of Nottingham, Nottingham, UK; 9 Nottingham NIHR Biomedical Research Centre, Faculty of Medicine and Health Sciences, University of Nottingham, Nottingham, UK; 10 Department of Twin Research & Genetic Epidemiology, King’s College London, London, UK

**Keywords:** Longitudinal, Incidence, Progression, Prediction trauma, Serum biomarkers, Pain, ADVANCE

## Abstract

**Objective:**

Knee osteoarthritis (OA) is a leading cause of disability globally, with previous injury a key risk factor. This study investigates the relationship between the risk of progression and incidence of knee radiographic OA (rOA) and pain (KP) with major traumatic injury and identify potential predictors for rOA and KP.

**Design:**

The longitudinal ADVANCE cohort study observes UK male military personnel, n=579 sustained combat-injury and n=565 uninjured, frequency-matched for age, rank, role, service, and deployment, in Afghanistan (2003-2014). Participants had bilateral radiographs (unless amputated), pain questionnaires, six-minute walk-tests, and serum collected, 8- and 11-years post-injury (Baseline and Follow-up). We compared rates and risk of rOA and KP incidence and progression and assessed the performance of twenty potential demographic, joint-specific and molecular predictors.

**Results:**

Knee rOA and KP incidence and progression rates increased between Baseline and Follow-up. rOA progression did not differ between trauma-exposed and unexposed groups; however, KP progression increased for those sustaining a specific knee injury (IRR:2.53, 95% CI:1.08-5.92. Increased rOA incidence was seen in those with lower-limb loss (IRR:1.98, 95% CI:1.21-2.95), and KP incidence in trauma-exposed participants with no limb-loss or knee injury (IRR:1.45, 95% CI:1.05-2.01). No predictive model explained a large proportion of variance for rOA or KP incidence or progression.

**Conclusions:**

Our results are consistent with a short post-injury window of increased rOA incidence, followed by a plateau. Comparatively, individuals with lower-limb loss experience a similar increase due to trauma, and then continue a steeper decline in joint-health due to altered joint biomechanics and mechanoinflammation.

**Supplementary Information:**

The online version contains supplementary material available at 10.1186/s13075-026-03739-4.

## Background

Globally, osteoarthritis (OA) is most prevalent at the knee [1, 2], accounting for up to 85% of OA burden [3], with knee OA incidence risk highest between 50- and 75-years old [4]. However, previous knee injury, female sex, obesity, high-impact sports, manual occupation, and genetics also increase the risk of knee OA [2, 5]. Current management of established OA focusses on symptomatic improvement, with pain the most disabling symptom and end-stage disease requiring surgical interventions [2]. OA is diagnosed using patient-reported and clinical features and imaging; however, there is a new focus on identifying early-stage knee OA [6], particularly recognising the factors predicting OA development to enable earlier intervention [7, 8].

Existing literature demonstrated a heightened risk of OA after an injury. The UK Biobank suggests time-bound increased OA progression following traumatic knee injury before levelling-out [9], indicating a brief window for potential intervention. This finding is replicated in other populations, including the military and professional athletes. In the Armed Forces, there is increased OA risk between 2- and 8-years following combat and musculoskeletal injury [10–12], and also in ex-footballers, with an initial increase in OA risk seen, before subsequently plateauing [13]. In those sustaining significant trauma, such as combat-injury, PTOA presentation is accelerated, with symptoms developing within two years [11]. However, there is an evidence gap regarding the longer-term implications of these injury types. This gap is especially relevant given that individuals with lower-limb loss have a fourfold increased odds of knee OA compared to matched controls [14] and evidence of longitudinal progression in the intact-side knee [15]. Ongoing surveillance of knee OA in these individuals is an important clinical concern.

The longitudinal ADVANCE cohort study is investigating physical and psychosocial outcomes of combat trauma following the Afghanistan war [16]. Earlier work in this cohort demonstrated those sustaining combat-injury had twofold greater odds for having knee radiographic OA (rOA) 8-years post-injury, with those sustaining a lower-limb loss or knee-specific injury having fourfold greater odds compared to uninjured participants [12]. Molecular biomarkers were not able to differentiate between those developing OA in trauma-exposed (post-traumatic OA, PTOA) or unexposed (idiopathic OA) groups, potentially suggesting that by 8-years after injury, both processes share a convergent common pathomechanistic pathway [17].The completion of the first ADVANCE Follow-up visit (11-years post-injury), approximately 3-years after Baseline data collection (8-years post-injury), offers the opportunity to compare two-wave trends for rOA and knee pain incidence and progression and predict the progression of knee rOA or pain.

Therefore, the primary aim of this study is to investigate the relationship between injury and the risk of progression and incidence of knee rOA and pain. We hypothesised that there will be (i) no increased risk of progression and incidence for trauma-exposed participants compared to unexposed participants between Baseline and Follow-up, but (ii) an increased risk of progression and incidence for those with lower-limb loss compared to unexposed participants. Our secondary aim was to assess the predictive value of pre-selected characteristics for the progression and incidence of knee rOA and pain.

## Methods

### Ethics

Ethical approval was provided by the Ministry of Defence (357.PPE/12) with additional approval from the University of Nottingham (FMHS 170-1122). Participants provided voluntary written informed consent at each visit.

### Study design & participants

ADVANCE involves 579 male participants who sustained combat injuries requiring aeromedical evacuation to the UK and hospital admission (Exposed), who are compared to 566 participants not exposed to combat injury, frequency-matched for age, rank, role, service, and deployment (Unexposed) [16]. Public and patient involvement is performed with participant focus-groups, newsletters, impact reports and website (www.advancestudydmrc.org,uk), refining study design, research outcomes and future care.

### Groups

Participants were divided into Unexposed and Exposed groups, as described above. The Exposed group were further sub-divided into:

1. Knee Injured (Exp-K),
2. Lower Limb Amputation (Exp-A),
3. No Lower Limb Amputation (Exp-NA).

The Exp-K group contained participants with and without lower-limb loss who sustained knee-specific combat injury (ICD-10:S80-89; ‘injury to the knee and lower leg’ or manual categorisation as local knee injury) recorded in contemporaneous records to isolate the known effect of traumatic knee injury on OA [18]. Such injuries include: knee dislocations, fractures to the proximal tibia, patella, distal femur and tibial plateau, cruciate ligament rupture, and meniscal tears. When both a knee injury and lower-limb amputation were present, they were coded as Exp-K, in line with previous analyses, as the knee injury is hypothesised to be more severe [12, 17].

**Fig. 1:**
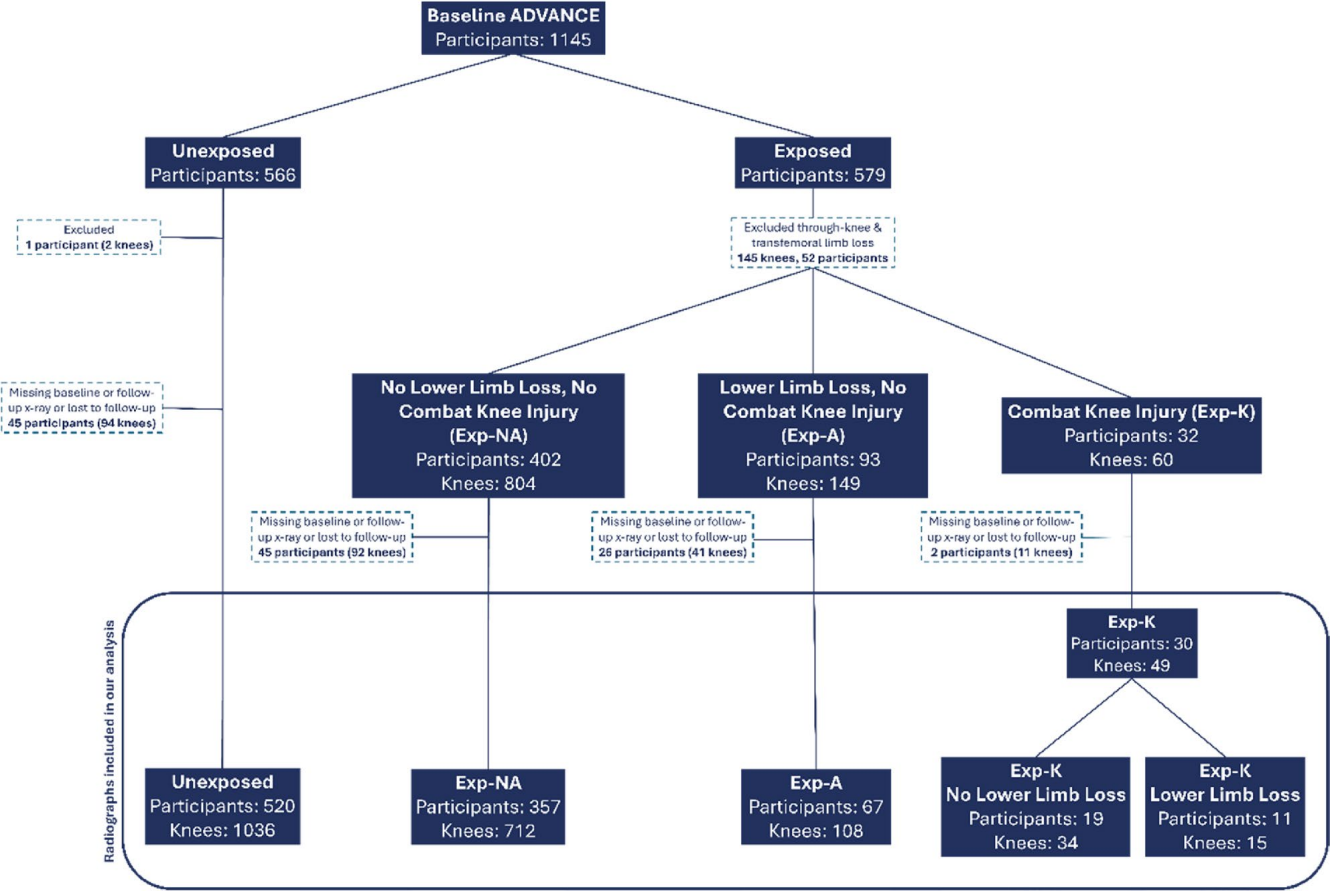
This diagram shows how many participants and knees were considered in the analysis for radiographic knee osteoarthritis progression or incidence. For inclusion in the analysis, successfully scored baseline and follow-up radiographs were required. For a detailed account of how many participants were lost to follow-up, and reason for missing radiographs and baseline and follow-up, please see Supplementary File E. N.B. 1 participant does not equate to 2 knees because a single knee radiograph can be lost or a lower-limb could have been amputated at a level above the knee, without losing a participant

The Exp-A group contained participants with both unilateral and bilateral lower-limb loss (Fig. 1), with a variety of amputation levels and combinations. Knees described are from both the intact- and amputation-side limbs, where a knee is present.

### Data collection

Data were collected at the Defence Medical Rehabilitation Centre Headley Court (2015–2018) and Stanford Hall (2018–2024). Each visit required one day of comprehensive, nurse-led assessments using REDCap [19], described comprehensively elsewhere [16]. Briefly, these included demographic details, medical history of combat-injuries (including severity, via New Injury Severity Scale; NISS), patient-reported outcome measures (PROMs), the six-minute walk-test (6MWT), knee radiographs and serum biomarkers [20–22].

To determine any pre-existing knee pathology before study recruitment, with an increased risk of OA, self-reported medical history and electronic health records were reviewed.

For participants with lower-limb loss, height was measured directly wearing prosthetics or pre-limb-loss values were recorded. Weight was measured without prostheses, and an adjusted weight was calculated depending on the level and limb/s missing, with a corresponding adjusted body mass index (BMI) [23].

### Self-reported questionnaires

#### KOOS

The KOOS is a widely used, reliable, and valid PROM, with five independent subscales scored 0-100 (100 denotes no knee problems) [21, 24, 25]. The Pain subscale was used to determine the presence of significant knee pain with a threshold of ≤ 86.1 [26, 27] and minimum clinically important difference (MCID) of 12.4 [28]. Knee pain Progression was defined as a decrease in intra-knee KOOS Pain score at Follow-up of ≥12.4 than at Baseline. Incidence of knee pain was defined as a KOOS Pain score ≤ 86.1 at Follow-up after a KOOS Pain score of > 86.1 in the same knee at Baseline.

#### KHJP

The KHJP score comprises three, 0 (no pain) to 10 (worst pain imaginable) Likert pain scales for severity, frequency, and impact for each knee and hip joint. Only knee scores were considered here.

### Radiographic assessment

Semi-flexed posterior-anterior radiographs were taken as previously described [16, 17]. The Kellgren-Lawrence (KL) score, graded between 0 (none) and 4 (severe), and Osteoarthritis Research Society International (OARSI) scores for joint space narrowing (JSN), between 0 (none) and 3 (severe), were assigned by the semi-automated Knee Osteoarthritis Labeling Assistant (KOALA) software, with a specificity of 88%, sensitivity of 78% and accuracy of 82% for KL grades [29–31].

Progression was defined as an increase in KL by ≥ 1 from Baseline to Follow-up in the same knee, with knee rOA (KL ≥ 1) present at Baseline. Knees with KL4 at Baseline were excluded from the Progression analysis due to a ceiling effect (*n* = 4 knees). Incidence was defined as the presence of knee rOA (KL ≥ 1) at Follow-up in a knee that was KL0 at Baseline.

### Baseline biomarker analysis

Biomarker sampling and analysis have been previously described [17]. Briefly, fasted blood was collected at Baseline, centrifuged (3500 rpm for 10 min), aliquoted and frozen at – 80 °C before transfer to Affinity Biomarker Labs (London, UK). Using Enzyme-Linked Immunosorbent Assay (ELISA) or Meso Scale Discovery (MSD), samples were analysed for metabolic (adiponectin, leptin), inflammatory (interleukin, IL-1β, -17α, tumour necrosis factor-alpha, TNF-α) and extracellular matrix (ECM) turnover (cartilage oligomeric protein, COMP, N-propeptide of collagen IIA, PIIANP, and C-terminal cross-linked telopeptide of type II collagen, CTX-II) biomarkers, on plates containing kit and quality control samples. Individual serum biomarker concentrations, intra- and inter-variability coefficient of variation and quantification concentration thresholds are found in Supplementary File A and here [17].

### Statistical analysis

Of 1145 participants, 1 Unexposed participant was excluded due to non-combat trauma lower-limb loss following matched deployment, and 92 participants were excluded because they did not attend Follow-up (attrition rate 8.04%). Radiographic data was available at Baseline and Follow-up for 1905 knees (974 participants)(Fig. 1). Further details regarding reasons for missing data are contained in Supplementary File B. Pain data was available for 1796 knees (909 participants). Knee and participant numbers do not match due to retaining of participants with single knee data due to invalid questionnaires, radiographs, or limb-loss. Data were screened for normality visually using histograms and Q-Q plots, with parametric and non-parametric reporting measures and testing used accordingly. Statistical analysis were performed on StataSE 18.1 (StataCorp LLE, Texas).

N.B. 1 participant does not equate to 2 knees because a single knee radiograph can be lost or a lower-limb could have been amputated at a level above the knee, without losing a participant.

Knee rOA and pain Progression and Incidence rates were calculated for each knee. A mixed-effects Poisson regression model appropriate for count data, accounting for the correlation between knees, with robust standard errors, was used to assess the risk of Progression and Incidence of knee rOA and Pain in Unexposed vs. Exposed, and Unexposed vs. Exp-NA/Exp-A/Exp-K groups. Results are reported by exposure group, followed by injury group. Models were adjusted for age at Baseline, time interval, body mass, smoking and socioeconomic status (SES)(using military rank as a proxy for National Statistics socioeconomic classification) [32, 33]. Risk Ratios (RR) and 95% Confidence Intervals (CI) are reported. A sensitivity analysis was conducted to test for potential bias resulting from inclusion of an individual’s left and right knees because they are not considered independent. Regression models were run for all knees, then separately for left and right knees, and results compared (Supplementary File C).

Presence of knee rOA or pain at Baseline was used to dichotomise individuals for predictive modelling, due to the previous similarities in molecular patterns between iOA and PTOA [17]. Twenty potential predictor variables were selected, based on their known risk for OA development or earlier work in the cohort: age, BMI, SES, NISS, time from injury, KOOS Pain & Symptom, knee pain impact, frequency, and severity, JSN, 6MWT distance (6MWD) and serum biomarkers [2, 7, 9, 10, 17]. JSN was selected given its use as an endpoint in clinical trials [34].

Predictor variables were combined into three models to assess their ability to predict knee rOA and pain progression and incidence at Follow-up. These three models, and their constituents, were;

- Model 1: Demographic (including injury-related and functional; age, BMI, NISS, time from injury, 6MWD)
- Model 2: Joint-specific (JSN, KOOS and knee pain scores), and
- Model 3: Molecular (serum biomarkers).

Initially, Spearman’s correlations were performed between the outcome and predictor variables as part of the model building. Subsequently, multivariable logistic regression was performed with each model, with Nagelkerke’s R^2^ and area under the receiver operator curve (AUROC) reported. The main analyses were performed with the Exp-A group excluded.

A separate Exp-A sub-group analysis was then performed. Due to the smaller numbers in Exp-A group preventing a similar approach outlined above, a Least Absolute Shrinkage and Selection Operator (LASSO) variable selection model was used. LASSO identified any significant predictor variables, with only one outcome (Pain incidence) resulting in a predictive model. All other outcomes could not be predicted.

## Results

1052 participants attended Baseline and Follow-up data collections (mean interval: 40.2-months, SD: 6.8-months). The retention rate was 92.0%; 93.1% (526/565) in the Unexposed group and 90.9% (526/579) in the Exposed group. Of those 92 participants lost to follow-up, *n* = 13 (17.1%) Unexposed and *n* = 17 (16.3%) Exposed participants had KL ≥ 1 at Baseline. At Follow-up, mean age was 38.2-years (SD: 5.4-years), further demographic characteristics can be found in Table 1.

**Table 1:** Participant demographics at follow-up for participants who attended baseline and follow-up data collection (*n* = 1052) in the unexposed and exposed groups, plus exposed sub-groups, Exp-K, Exp-NA, and Exp-A

	Unexposed (*n* = 526)	All Exposed (*n* = 526)	Exp-NA (*n* = 371)	Exp-A (*n* = 122)	Exp-K (*n* = 33)
Age (years)	38.3 (5.4)	38.0 (5.3)	38.3 (5.5)	37 (4) 4.8)	37.6 (5.7)
Time since baseline (years)	3.4 (0.6)	3.3 (0.6)	3.3 (0.5)	3.5 (0.6)	3.3 (0.5)
Previous knee injury (Yes%)	27 (5.1)	10 (1.9)	9 (2.4)	1 (0.8)	0 (0.0)
Cause of injury					
Blast		360 (68.4)	218 (58.8)	118 (96.7)	24 (72.7)
Gunshot	-	124 (23.6)	112 (30.2)	4 (3.3)	8 (24.2)
Other		2 (0.4)	1 (0.3)	0 (0.0)	1 (3.0)
		40 (7.6)	40 (10.8)	0 (0.0)	0 (0.0)
Height (cm)	180.0 (6.3)	179.5 (6.9)	179.1 (6.7)	180.7 (7.6)	179.2 (6.8)
Mass* (kg)	90.3 (13.0)	91.5 (15.1)	90.8 (14.9)	93.2 (15.6)	92.5 (15.5)
BMI* (kg/m^2^)	28.2 (3.7)	28.5 (4.1)	28.3 (3.9)	30.0 (4.5)	28.9 (4.6)
Race (White)	470 (89.4)	470 (89.4)	331 (89.2)	107 (87.7)	32 (97.0)
NISS (median, 25th – 75th percentile)	-	12 (5–22)	9 (4–17)	22 (14–34)	13 (12–22)
NC-SEC					
Officer rank	75 (14.3)	58 (11.0)	44 (11.9)	12 (9.8)	2 (6.1)
Senior-rank	142 (27.0)	100 (19.0)	79 (21.3)	16 (13.1)	5 (15.2)
Junior rank	309 (58.8)	368 (70.0)	248 (66.9)	94 (77.1)	26 (78.8)
Still serving in military (yes)	355 (67.9)	109 (20.8)	104 (28.2)	3 (2.5)	2 (6.1)

Age at follow-up, time since baseline, time between injury and assessment, height, mass, and BMI are reported as mean (SD). The remaining variables are reported as count (percentage).

*Mass and BMI are both adjusted for limb loss as described in Methods

Thirty-seven individuals had pathology before index injury/deployment increasing the risk of OA (fracture, cruciate, meniscal). Chi-squared test revealed no significant differences between the Unexposed and Exposed groups, negating any effect of prior injury.

**Fig. 2:**
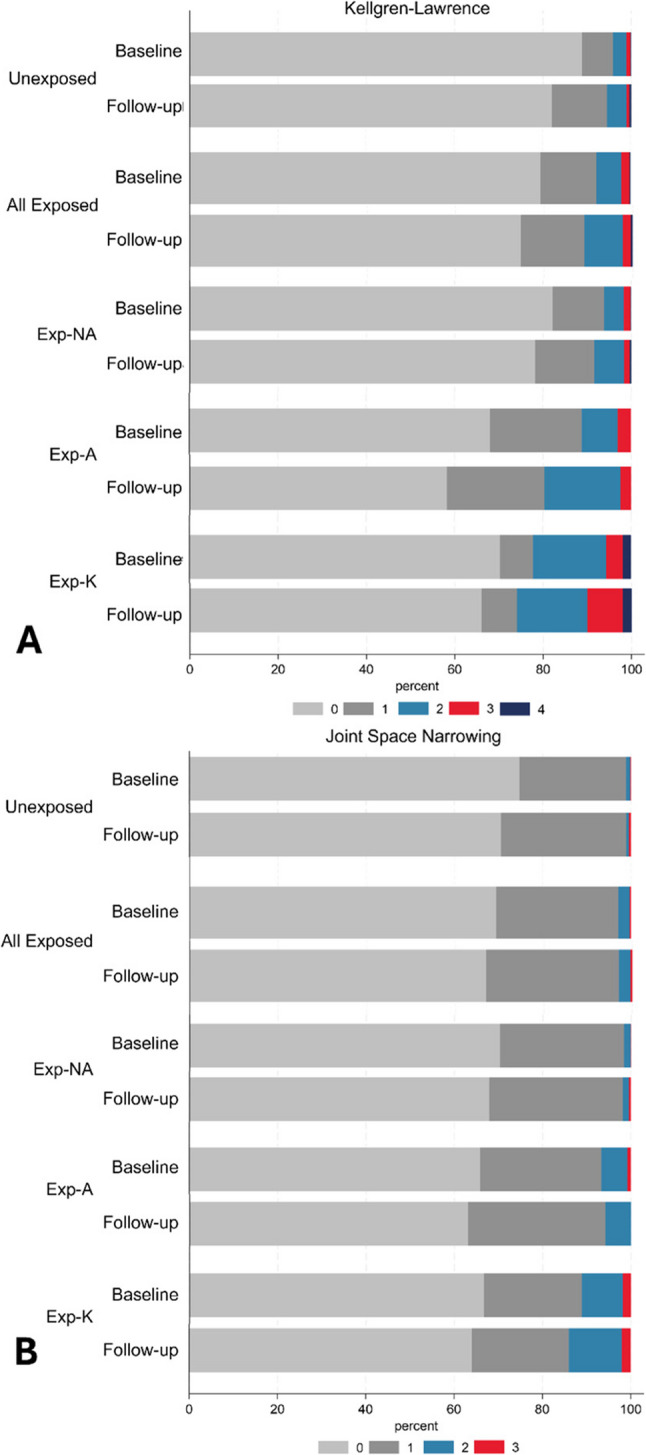
Kellgren-Lawrence (KL; [**A**]) and Joint Space Narrowing (JSN; [**B**]) scores for radiographic knee osteoarthritis of participants across unexposed, Exp-NA, Exp-A, and Exp-K groups with Kellgren-Lawrence at baseline and follow-up

**Fig. 3:**
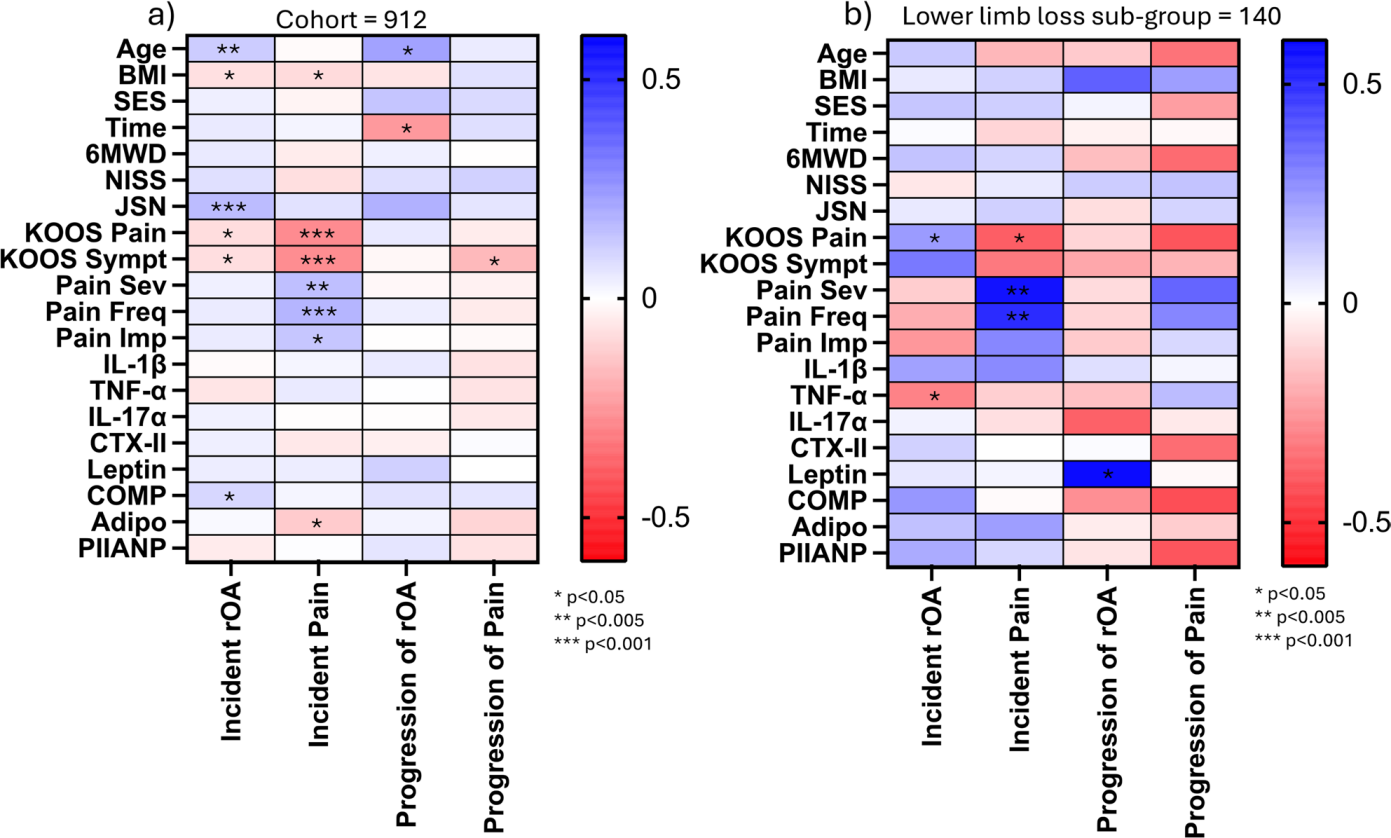
Correlations between potential predictor variables and incidence or progression of radiographic OA and KOOS pain in the whole cohort excluding those with lower-limb loss (**a**) and lower-limb loss sub-group (**b**). JSN: Joint Space Narrowing, KOOS: Knee injury and Osteoarthritis Outcome Score, Sympt: Symptom, Sev: Severity, Freq: Frequency, Imp: Impact, rOA: Radiographic Osteoarthritis, L: Left, R: Right, BMI: Body mass index, SES: Socioeconomic status, 6WMD: Six minute walk distance, NISS: New injury severity score, IL: Interleukin, TNF: Tumour necrosis factor, CTX-II: C-terminal cross-linked telopeptide of type II collagen, COMP: Cartilage oligomeric protein, PIIANP: N-propeptide of collagen IIA. Test used: Spearman’s correlation. Not all participants had complete data for all variables, correlations were performed per variable with complete data to avoid unnecessary data exclusion. *p<0.05 **p<0.005 ***p<0.001

KL, JSN and KOOS Pain scores at Baseline and Follow-up are reported in Table 2; Fig. 2, and Supplementary File D. Correlation results are summarised in Fig. 3 (full results, Supplementary File E) with the regression results reported in the text (full results, Supplementary File F).

**Table 2:** Radiographic (Kellgren-Lawrence [KL] and Joint Space Narrowing [JSN]) and self-reported (Knee Osteoarthritis Outcome Score [KOOS] Pain) measures of knee osteoarthritis at Baseline (BL) and Follow-up (FU) for the unexposed and exposed groups, as well as the exposed sub-groups (Exp-K, Exp-NA, Exp-A)

Radiographic Knee Osteoarthritis										
	Unexposed (*n* = 1036)		Exposed (*n* = 869)		Exp-NA (*n* = 712)		Exp-A (*n* = 108)		Exp-K (*n* = 49)	
	BL	FU	BL	FU	BL	FU	BL	FU	BL	FU
KL										
0	922 (89.0)	851 (82.1)	693 (79.8)	654 (75.3)	587 (82.4)	558 (78.4)	72 (66.7)	64 (59.3)	34 (69.4)	32 (65.3)
1	75 (7.2)	128 (12.4)	109 (12.5)	121 (13.9)	82 (11.5)	94 (13.2)	23 (21.3)	23 (21.3)	4 (8.2)	4 (8.2)
2	29 (2.8)	46 (4.4)	51 (5.9)	74 (8.5)	33 (4.6)	48 (6.7)	10 (9.3)	18 (16.7)	8 (16.3)	8 (16.3)
3	8 (0.8)	6 (0.6)	14 (1.6)	16 (1.8)	9 (1.3)	9 (1.3)	3 (2.8)	3 (2.8)	2 (4.1)	4 (8.2)
4	2 (0.2)	5 (0.5)	2 (0.2)	4 (0.5)	1 (0.1)	3 (0.4)	0 (0.0)	0 (0.0)	1 (2.0)	1 (2.0)
JSN										
0	777 (75.0)	732 (70.7)	596 (68.6)	579 (66.6)	493 (69.2)	482 (67.7)	71 (65.7)	66 (61.1)	32 (65.3)	31 (63.3)
1	249 (24.0)	293 (28.3)	251 (28.9)	263 (30.3)	209 (29.4)	217 (30.5)	31 (28.7)	35 (32.4)	11 (22.5)	11 (22.5)
2	8 (0.8)	6 (0.6)	20 (2.3)	23 (2.7)	9 (1.3)	10 (1.4)	6 (5.6)	7 (6.5)	5 (10.2)	6 (12.2)
3	2 (0.2)	5 (0.5)	2 (0.2)	4 (0.5)	1 (0.1)	3 (0.4)	0 (0.0)	0 (0.0)	1 (2.0)	1 (2.0)

**Knee Osteoarthritis Outcome Score (KOOS)**										
	**Unexposed** **(** ***n*** ** = 966)**		**Exposed** **(** ***n*** ** = 830)**		**Exp-NA** **(** ***n*** ** = 669)**		**Exp-A** **(** ***n*** ** = 106)**		**Exp-K** **(** ***n*** ** = 55)**	
	**BL**	**FU**	**BL**	**FU**	**BL**	**FU**	**BL**	**FU**	**BL**	**FU**
KOOS Pain	100 (89–100)	100 (89–100)	97 (81–100)	97 (81–100)	97 (83–100)	100 (81–100)	97 (83–100)	97 (86–100)	86 (67–100)	86 (58–100)

KL and JSN are presented as number, *n*= (percentage, %)

KOOS Pain is presented as median (interquartile range)

*N * relates to number of knees

### Progression

#### Radiographs

Out of 1,905 knees (Fig. 1 and Supplementary File D), 286 (15%) had KL ≥ 1 at Baseline; 46 (16.1%) had progression at Follow-up. Radiographic progression occurred in 19 (17.0%) of Unexposed, 16 (12.3%) of Exp-NA, 7 (19.4%) of Exp-A and 4 (28.6%) of Exp-K participants. There was no increased rOA progression risk in the Exposed compared to the Unexposed group (RR 0.98, 95% CI:0.57–1.68, Table 3). Subgroup analysis revealed no significantly different risk of rOA progression in the Exp-A (RR 1.21, 95% CI:0.56–2.60) and Exp-K (RR 1.65, 95% CI:0.66–4.14) groups (Table 3) compared to the Unexposed group.

**Table 3:** Incidence Rate Ratio (IRR) for radiographic knee OA progression (rOA) and KOOS pain score progression between baseline and follow-up. Analyses were adjusted for baseline age, interval between baseline and follow-up data collection, smoking status, BMI, and socioeconomic status

rOA progression			
Group	Unadjusted	Adjusted	
	IRR (95% CI)	IRR (95% CI)	*p* value
Exposure status			
Unexposed (*n* = 112)	1 (ref)	1 (ref)	0.933
Exposed (*n* = 174)	0.92 (0.53–1.58)	0.98 (0.57–1.68)	
Injury status			
Unexposed (*n* = 112)	1 (ref)	1 (ref)	0.430
Exp-NA (*n* = 124)	0.76 (0.41–1.40)	0.84 (0.46–1.54)	
Exp-A (*n* = 36)	1.15 (0.52–2.52)	1.21 (0.56–2.60)	
Exp-K (*n *= 14)	1.68 (0.70–4.06)	1.65 (0.66–4.14)	

**KOOS Pain progression**			
**Group**	**Unadjusted**	**Adjusted**	
	**IRR** **(95% CI)**	**IRR** **(95% CI)**	***p*** **value**
Exposure status			
Unexposed (*n* = 236)	1 (ref)	1 (ref)	0.194
Exposed (*n* = 284)	1.35 (0.78–2.34)	1.44 (0.83–2.51)	
Injury status			
Unexposed (*n* = 236)	1 (ref)	1 (ref)	0.171
Exp-NA (*n* = 218)	1.29 (0.71–2.35)	1.38 (0.76–2.50)	
Exp-A (*n* = 36)	0.83 (0.20–3.46)	0.90 (0.21–3.89)	
Exp-K (*n* = 30)	2.42 (1.05–5.59)	2.53 (1.08–5.92)	

*rOA* radiographic osteoarthritis, *KOOS *Knee injury and osteoarthritis outcome score, *IRR* incidence rate ratio, *CI* Confidence interval, *Exp-NA* Exposed, no lower-limb amputation, *Exp-A* Exposed, lower-limb amputation, *Exp-K* Exposed, knee injured

Sensitivity analysis showed no difference for results reporting left knee only, right knee only, or both knees together.

Predictive analysis demonstrated that Model 1 had an AUROC 0.80 (0.67,0.92); Model 2, 0.72 (0.61,0.83); and Model 3, 0.66 (0.55,0.77) for predicting rOA progression. The highest R^2^ was 0.22 for Model 1.

### KOOS pain progression

Out of 1,796 knees, 520 (29%) had a KOOS Pain score ≤ 86.2 at Baseline; 61 (11.7%) showed pain progression at Follow-up. Pain progression occurred in 23 (9.8%) of Unexposed, 28 (12.8%) of Exp-NA, 3 (8.8%) of Exp-A and 7 (24.1%) of Exp-K participants. The risk for Pain progression was not different for the Exposed group compared to the Unexposed group (RR 1.44, 95% CI:0.83–2.51), or for the Exp-NA or Exp-A groups compared to the Unexposed group (RR 1.38, 95% CI:0.76–2.50; RR 0.90, 95% CI: 0.21–3.89), but was 2.53 (95% CI:1.08–5.92) times higher for the Exp-K group compared to the Unexposed group (Table 3).

Predictive Model 1 had an AUROC 0.62 (0.50,0.75); Model 2, 0.70 (0.58,0.76); and Model 3, 0.62 (0.52,0.71) for the prediction of Pain progression. Model 2 had the highest R^2^, 0.07.

### Incidence

#### Radiographs

One thousand six hundred fifteen knees were KL0 at Baseline, of which 189 (11.7%) had KL ≥ 1 at Follow-up. Radiographic incidence occurred in 103 (11.2%) of Unexposed, 65 (11.1%) of Exp-NA, 17 (23.6%) of Exp-A and 4 (11.8%) of Exp-K participants. Knee rOA RR was not different for the Exposed group compared to the Unexposed group (RR 1.09, 95% CI:0.82–1.45, *p* = 0.560), but the Exp-A group had a 1.89 (95% CI:1.21–2.95, *p* = 0.007) times higher risk than the Unexposed group (Table 4).

**Table 4:** Incidence rate ratios for incidence of radiographic knee OA and KOOS pain score between baseline and Follow-up. Adjusted models were adjusted for baseline age, interval between baseline and follow-up data collection, smoking status, BMI, and socioeconomic status

rOA incidence			
Group	Unadjusted	Adjusted	
	IRR (95% CI)	IRR (95% CI)	*p* value
Exposure status			
Unexposed (*n* = 922)	1 (ref)	1 (ref)	0.560
Exposed (*n* = 693)	1.11 (0.83–1.49)	1.09 (0.82–1.45)	
Injury status			
Unexposed (*n* = 922)	1 (ref)	1 (ref)	0.031
Exp-NA (*n* = 587)	0.99 (0.72–1.36)	0.99 (0.72–1.35)	
Exp-A (*n* = 72)	2.11 (1.25–3.55)	1.89 (1.21–2.95)	
Exp-K (*n* = 34)	1.06 (0.39–2.89)	1.03 (0.40–2.70)	

**KOOS Pain Incidence**			
**Group**	**Unadjusted**	**Adjusted**	
	**IRR** **(95% CI)**	**IRR** **(95% CI)**	***p*** **value**
Exposure status			
Unexposed (*n* = 730)	1 (ref)	1 (ref)	0.042
Exposed (*n* = 548)	1.49 (1.08–2.04)	1.39 (1.01–1.90)	
Injury status			
Unexposed (*n *= 730)	1 (ref)	1 (ref)	0.126
Exp-NA (*n* = 451)	1.53 (1.10–2.13)	1.45 (1.05–2.01)	
Exp-A (*n* = 72)	1.11 (0.54–2.32)	0.96 (0.48–1.92)	
Exp-K (*n* = 25)	1.77 (0.75–4.19)	1.40 (0.62–3.17)	

*rOA* radiographic osteoarthritis, *KOOS* Knee injury and osteoarthritis outcome score, *IRR* incidence rate ratio, *CI* Confidence interval, *Exp-NA* Exposed, no lower-limb amputation, *Exp-A* Exposed, lower-limb amputation, *Exp-K* Exposed, knee injured

Model 1 had an AUROC 0.66 (0.57,0.74); Model 2, 0.63 (0.57, 0.68); and Model 3, 0.58 (0.53,0.64) for the prediction of rOA incidence. Model 1 had the highest R^2^, 0.06.

### KOOS pain

1278 knees had a KOOS Pain score > 86.2, of which 181 (14.2%) reported new pain (≤ 86.1) at Follow-up. Incident pain occurred in 86 (11.8%) of Unexposed, 81 (18.0%) of Exp-NA, 9 (13.0%) of Exp-A and 5 (20.0%) of Exp-K participants. The RR for Pain incidence was 1.39 × (95% CI:1.01–1.90, *p* = 0.042) higher for the Exposed group compared to the Unexposed group, with the Exp-NA group having a 1.45 × (95% CI:1.05–2.01, *p* = 0.018) higher risk for Pain incidence than the Unexposed group (Table 4).

Model 1 had an AUROC 0.69 (0.61,0.76); Model 2, 0.70 (0.64,0.76); and Model 3, 0.60 (0.54,0.65) for the prediction of knee Pain incidence. Model 1 and 2 both had the highest R^2^ (0.08). Within the Exp-A sub-group, knee pain frequency and severity were selected by LASSO, creating a model with AUROC 0.83, R^2^ 0.33.

## Discussion

This large, unique study describes the progression and incidence of knee rOA and pain in a military cohort 8- and 11-years after severe combat injury. We demonstrate novel findings related to rOA incidence in those with traumatic lower-limb loss and the value of a pre-selected panel of Baseline variables to predict radiographic or symptomatic change. Most significantly, participants with a lower-limb amputation had a nearly twofold greater risk of developing knee OA, those with a knee injury had a 2.5x risk of worsening pain, and with the remaining injured participants had a 1.5x risk of new pain.

### Progression and incidence

In keeping with our hypothesis, no Exposed subgroup showed significantly different risk for rOA progression compared to the Unexposed group in the 3.3-years between Baseline and Follow-up. This was based on previous work demonstrated that knee rOA and pain were both significantly worse in the Exp-NA, Exp-A and Exp-K groups compared to the Unexposed group [12]. However, the Exp-K group showed a 2.52 times greater risk for Pain progression compared to the Unexposed group.

The lack of different rOA progression between groups mirrors other cohort studies [9, 13]. Within the UK BioBank, involving over 500,000 individuals, the risk of OA for those with a traumatic knee injury, compared to controls, peaked 5-years post-injury, after which it decreased gradually [9]. Our findings also suggest that people with a traumatic knee injury have higher initial risk rates of rOA initially, but after 8-years, it progresses at a similar rate to the Unexposed.

Despite no increased rOA progression risk in the Exp-K group, they did have a 2.52-fold greater risk of knee Pain progression that the Unexposed participants, with earlier work showing they also had significantly worse Pain scores at Baseline [12]. It is possible that the Exp-K group have undergone peripheral pain sensitisation, with recent work in those with knee OA showing people with high symptom severity often had lower physical pain thresholds than people with low symptom severity [35]. The median KOOS Symptoms score for the Exp-K group at Follow-up was more severe than all other participants, potentially contributing to a lower pain threshold and exaggerating the risk of pain progression in the absence of rOA progression. Alternatively, this increased pain could be an early signal of future OA-associated disability, with accompanying structural changes not yet visible on radiographs. End-stage disease or overt disability would not be expected in such a young cohort; however, as the ADVANCE study progresses, it might become clearer that Exp-K individuals experience increased and earlier disability and end-stage disease compared to their peers.

There was a two times greater risk for rOA incidence in the Exp-A group compared to the Unexposed group at Follow-Up. It is possible that that the Exp-A group, alongside the other Exposed sub-groups, experienced an initial accelerated period of knee OA following trauma, perhaps within a similar 5-year window observed in the UK Biobank study [9, 36]. Subsequently, it is possible that altered biomechanics and mechanical overload become the primary OA pathomechanism, likely leading to the high rates of OA seen in people with lower-limb loss [14, 37]. Previous biomechanical studies have demonstrated increased joint contact forces through the medial compartment of the intact side limb [37], on which people with lower-limb loss preferentially rely [38], drive this elevated risk. As a result, this altered biomechanical environment may mean that people with limb-loss have a different disease trajectory from those who sustained other trauma, with trauma-induced OA developing in the injured side and biomechanically-driven OA in the contralateral limb initially. Further research is recommended to understand the contribution of unilateral/bilateral, level, and amputation/intact side of limb-loss, given their gait biomechanics differences [39], which would allow this aetiological relationship to be better explored.

Despite increased rOA incidence in the Exp-A group, there was no increased risk of pain incidence at the 11-year point. The dominant symptom of OA is pain, heavily influencing clinical decision-making [2]. Our findings suggest rOA with no/reduced pain, and therefore reduced opportunity for preventative management. Within this cohort, previous work demonstrated participants with lower-limb loss report less pain than those without [40], possibly related to the lower rates of anxiety and depression compared to the trauma-exposed participants without limb-loss [41]. Interestingly, the opposite was seen for the Exp-NA group; there was no increased risk for rOA progression or incidence but an increased risk for pain incidence at Follow-Up. Alternatively, these findings may be due to differences in central or peripheral pain processing in those with lower-limb loss. Clinically, new pain might stimulate early investigations or treatment for knee OA, with the opposite also true. Therefore, we recommend increased clinical vigilance for knee OA in people with lower-limb loss, as they may experience pain differently, or later; by which time opportunities for early intervention could be missed.

**Fig. 4:**
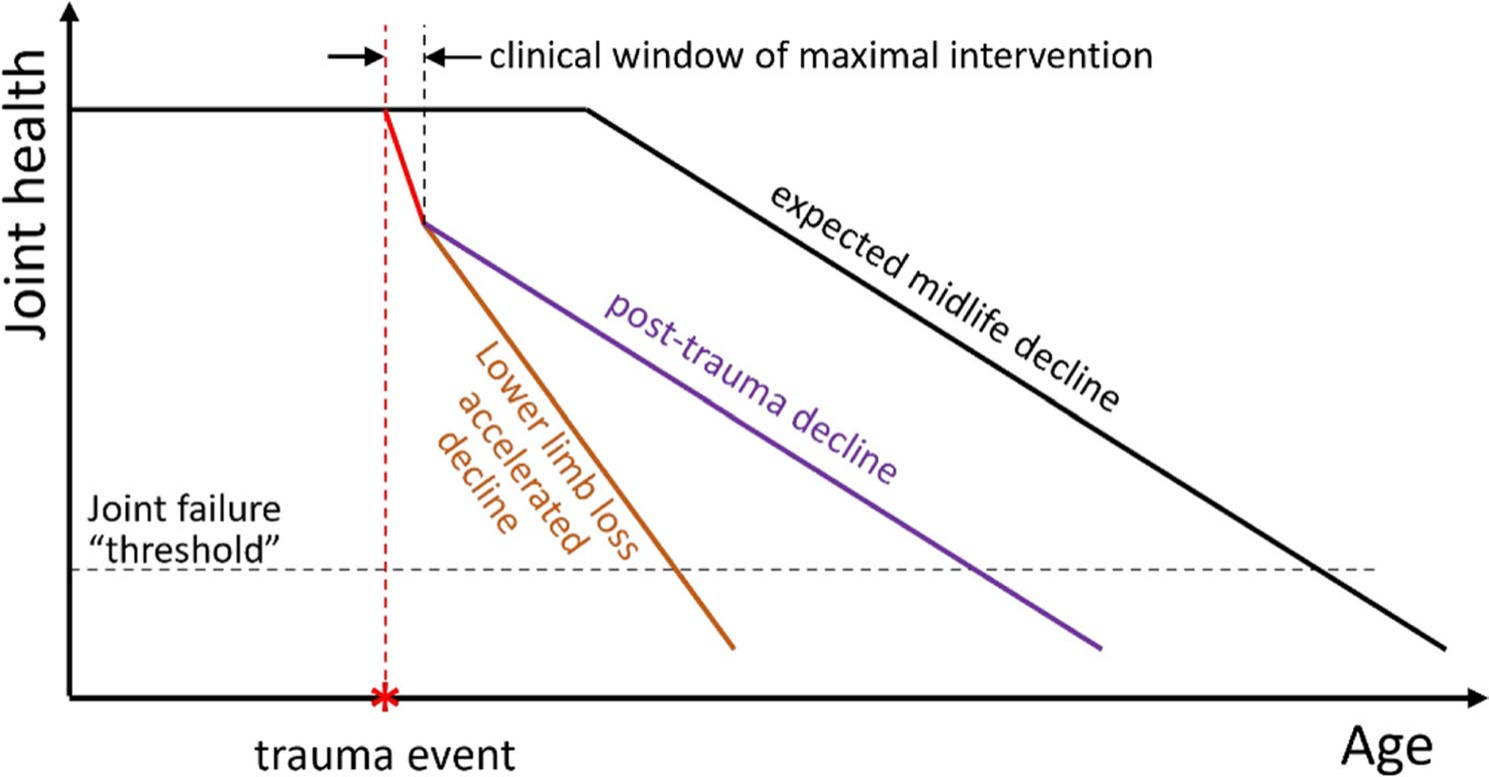
Theoretical model of joint health for healthy (solid line), traumatic injury (dashed line) and limb loss (dotted line) aetiologies. For all groups there is an assumed gradual reduction of joint health starting in midlife. Those with a traumatic injury and lower limb loss experience a steep decline in joint health post-injury. For those with a traumatic injury, this decline plateaus and returns to a similar rate of decline as a healthy joint, but those with limb loss continue to decline at a faster rate due to sustained mechanoinflammation resulting from altered joint biomechanics. The grey box represents with clinical window of maximal intervention

Based on these findings and existing literature, we present a new theoretical model for the potential trajectories of different OA aetiologies (Fig. 4). For a healthy population (solid line), there is a gradual reduction of joint health from approximately midlife. For post-traumatic groups (dashed line), there is a sharp decrease in joint health followed by a plateau and subsequent similar midlife decline. Finally, those with lower-limb loss (dotted line) experience a similar sharp decrease due to trauma, but then continue a steeper decline due to continued altered joint biomechanics and mechanoinflammation.

For those with a traumatic injury, including lower-limb loss, the initial increased risk of OA development infers a potential early ‘clinical window of maximal intervention’, supported by the negative correlation between time from injury and rOA development. These findings highlight the need for secondary prevention and timely intervention, commencing swiftly following injury or rehabilitation/recovery pathway completion, especially in high-risk populations [42, 43]. For those with lower-limb loss, this window for maximal intervention may be longer but more urgent because of accelerated development and should include interventions designed to normalise joint biomechanics.

### Predictive modelling

Identification of those at risk of knee rOA or pain can improve the success of any potential clinical interventions. These interventions, including physical activity (including cardiovascular, strengthening and neuromuscular exercises), appropriate weight loss, activity/job modification, and optimisation of nutrition and sleep, can improve OA outcomes, especially when targeted to the right person at the right time [42, 43]. In addition, enhanced participant identification and recruitment will improve pharmacological studies for disease-modifying therapies [42–45].

In this study, we used different models related to demographics, injury and function; joint-specific metrics; and molecular biomarkers to predict the incidence or progression of knee rOA or pain. Whilst overall, these combined models had reasonable AUROCs (between 0.58 and 0.80), the explanation of variance within the models was low (R^2^ between 0.02 and 0.22). Perhaps unsurprisingly, the model with the best fit was the demographic model (Model 1) for rOA progression, with an AIC of 77.8 and BIC of 93.3. It is clear that, similar to other populations [8, 9, 46], there remain challenges for predicting who will develop knee rOA or pain, especially when one notes the differing patterns seen across incidence and progression. This suggests different pathological progresses may be underway during development and maintenance of OA, requiring different predictive models.

Within the models, age and JSN had higher odds for rOA incidence; time from injury, JSN and knee pain frequency to rOA progression; BMI, KOOS Symptoms and knee pain frequency to Pain incidence and KOOS Symptoms to Pain progression (Supplementary File D). Perhaps it is self-evident that age would predict rOA, radiographic change would predict radiographic change, and pain would predict pain, but it remains important to include demographic, objective (radiological) and subjective (patient-reported) measures in future clinical risk prediction models or creation of OA phenotypes [5]. It was notable that BMI did not predict rOA risk. This might be explained by military personnel, similar to other athletic populations, having a higher percentage of muscle compared to adipose tissue [47].

From a molecular biomarker perspective, COMP was significant for rOA incidence, with adiponectin and CTX-II both having lower odds for Pain incidence (Supplementary File D). The most significant result, the relationship of adiponectin to pain, is consistent with previous results and literature; however, given the weak nature of the correlation and limited OR, the clinical significance is uncertain [17, 48]. Two possible explanations might contextualise the limited biomarker performance. Firstly, the significant period of time between injury and sampling – a previous review demonstrated biomarkers associated with structural or symptomatic change in the acute phase are less effective further from injury [49]. This suggests the current biomarkers may have initial value in determining elevated post-injury OA risk but may be less valuable when this risk reduces, with alternative metrics or biomarkers required [44, 45]. Another explanation might be the early stage of OA under investigation, with only 26 knees having KL3/4 at Baseline, rising to 31 by Follow-Up. It is possible that, given the small amount of severe disease, there is a limited systemic pattern, with the pathological signal still contained locally within the joint space, potentially only revealed by synovial fluid sampling.

There was a different picture in those with lower-limb loss (Fig. 3). During the pre-model stage, knee pain severity and frequency and KOOS Pain all correlated with incident Pain, with the latter also correlated with incident rOA. In addition, TNF-α correlated with incident rOA and leptin with rOA progression. However, no predictors for rOA incidence or progression were selected by LASSO, and those selected for incident Pain demonstrated non-significant OR. These results hint at the possible mechanoinflammation due to the ongoing biomechanical changes; however, they demonstrate that a different predictive paradigm is required for those with lower-limb loss.

### Limitations

This research utilises two-wave data from an ongoing longitudinal study, with only three or more waves of data capable of ascertaining patterns. Each prediction model only had a relatively small number of ‘events’, and therefore, our results should be interpretation with caution. Additionally, our definition of rOA progression required knees with a KL score of 4 to be excluded because there is no way for that score to increase/progress. This study has potential for survivor bias, so future longitudinal analysis should include all Baseline participants and account for missing data. Only a single radiographic view is reported, which might underreport the rates of rOA, with radiographic images also lacking the nuance of advanced imaging techniques such as magnetic resonance. With regards to comparability to other PTOA datasets, due to the nature of combat injury, ADVANCE participants are likely to have sustained much more serious and complex comorbid injuries that may affect rOA and their perception of pain in immeasurable ways. Also, female military personnel were excluded because the number sustaining combat injuries was too low to generate sufficient statistical power, therefore, validation is required in studies with both sexes. Finally, all molecular biomarkers were measured in a single form (serum), with floor and ceiling effects, which might affect their ability to detect changes.

## Conclusion

Male participants without lower-limb loss and with knee-specific combat trauma had the same rOA progression and incidence risk between 8- and 11-years post-injury as an unexposed group, having previously shown increased risk at 8-years. This suggests that a period of rapid PTOA progression may have already occurred, and a window of opportunity for maximal intervention passed – highlighting the need for early interventions in the immediate post-injury period. However, men with lower-limb loss had a sustained higher rOA incidence risk, perhaps due to continuous mechanical pathomechanism after the initial trauma. Despite this, they did not show an increased incidence of knee pain. Therefore, individuals with lower-limb amputation should be closely monitored for knee OA and opportunities for early intervention. Given the existing predictors and serum biomarkers did not explain much variance, further work is required to identify predictors which can enable the early recognition of those at risk of new or progressive knee rOA or pain, especially in the year after injury.

## Supplementary Information

Supplementary Material 1: Expanded Figure 1.

Supplementary Material 2: Sensitivity Analysis.

Supplementary Material 3: Correlation Analysis.

Supplementary Material 4: KOOS Pain Scores.

Supplementary Material 5: Predictive Modelling

Supplementary Material 6: Serum Biomarker Data.

## Data Availability

Data relate to serving and ex-serving military personnel, are sensitive and are not widely available, however, requests for data can be made via the corresponding author and will be considered on a case-by-case basis and subject to UK Ministry of Defence clearance. The code used for analysis will be shared on request to the corresponding author.
