# Supplementary material for: Influence of major trauma and lower limb loss on radiographic progression and incidence of knee osteoarthritis and pain: a comparative and predictive analysis from the ADVANCE study
Source: Arthritis Res Ther. 2026 Jan 26;28:49. doi: 10.1186/s13075-026-03739-4 (PMC12918490; doi:10.1186/s13075-026-03739-4)
Supplement: Supplementary file 2 — Supplementary Material 2: Sensitivity Analysis. [file 13075_2026_3739_MOESM2_ESM.docx]

Supplementary File C

1. Does considering all knees together for Unexposed vs. Exposed analysis of Kellgren-Lawrence progression affect the analysis outcome?

All knees

Right knees only

Left knees only

1. Does considering all knees together for Unexposed vs. Exposed analysis of Kellgren-Lawrence incidence affect the analysis outcome?

All knees

Right knees only

Left knees only

Summary

1.Progression – Left/Right

| **Test** | **IRR** | **CI** | ***p*-value** |
| --- | --- | --- | --- |
| Left & right knees | 1.06 | 0.62-1.82 | 0.840 |
| Left knees only | 1.16 | 0.53-2.56 | 0.707 |
| Right knees only | 1.07 | 0.48-2.42 | 0.866 |

2. Incidence – Left/Right

| **Test** | **IRR** | **CI** | ***p*-value** |
| --- | --- | --- | --- |
| Left & right knees | 1.11 | 0.83-1.48 | 0.494 |
| Left knees only | 0.99 | 0.68-1.43 | 0.942 |
| Right knees only | 1.25 | 0.85-1.85 | 0.257 |
