# Supplementary material for: Influence of major trauma and lower limb loss on radiographic progression and incidence of knee osteoarthritis and pain: a comparative and predictive analysis from the ADVANCE study
Source: Arthritis Res Ther. 2026 Jan 26;28:49. doi: 10.1186/s13075-026-03739-4 (PMC12918490; doi:10.1186/s13075-026-03739-4)
Supplement: Supplementary file 3 — Supplementary Material 3: Correlation Analysis. [file 13075_2026_3739_MOESM3_ESM.docx]

Supplementary file E. Correlation analysis of whole cohort with lower-limb loss subgroup excluded (a) and lower limb loss subgroup only (b) between twenty potential predictor variables to incidence and progression of knee radiographic osteoarthritis and pain

a)

| Cohort | Incident rOA | Incident Pain | Progression of rOA | Progression of Pain |
| --- | --- | --- | --- | --- |
| Age | **0.118** | -0.013 | **0.218** | 0.046 |
| BMI | **-0.075** | **-0.087** | -0.065 | 0.07 |
| SES | 0.037 | -0.028 | 0.135 | 0.085 |
| Time | 0.048 | 0.024 | **-0.24** | 0.075 |
| 6MWD | 0.048 | -0.047 | 0.035 | -0.003 |
| NISS | 0.072 | -0.077 | 0.074 | 0.108 |
| JSN | **0.157** | 0.067 | 0.182 | 0.059 |
| KOOS Pain | **-0.081** | **-0.274** | 0.05 | -0.047 |
| KOOS Sympt | **-0.078** | **-0.27** | -0.02 | **-0.167** |
| Pain Sev | 0.035 | **0.15** | -0.019 | -0.033 |
| Pain Freq | 0.046 | **0.172** | 0.04 | -0.049 |
| Pain Imp | 0.045 | **0.133** | -0.004 | -0.015 |
| IL-1β | -0.012 | 0.022 | 0.049 | -0.069 |
| TNF-α | -0.062 | 0.048 | 0.001 | -0.068 |
| IL-17α | 0.032 | -0.007 | -0.007 | -0.055 |
| CTX-II | 0.037 | -0.056 | -0.039 | 0.008 |
| Leptin | 0.042 | 0.042 | 0.11 | -0.002 |
| COMP | **0.094** | 0.023 | 0.067 | 0.059 |
| Adipo | 0.012 | **-0.124** | 0.028 | -0.101 |
| PIIANP | -0.048 | 0.003 | 0.06 | -0.07 |

b)

| Lower limb loss subgroup | Incident rOA | Incident Pain | Progression of rOA | Progression of Pain |
| --- | --- | --- | --- | --- |
| Age | 0.127 | -0.171 | -0.125 | -0.331 |
| BMI | 0.051 | 0.107 | 0.373 | 0.226 |
| SES | 0.133 | 0.112 | 0.024 | -0.23 |
| Time | 0.009 | -0.1 | -0.032 | -0.017 |
| 6MWD | 0.141 | 0.099 | -0.155 | -0.35 |
| NISS | -0.057 | 0.05 | 0.12 | 0.141 |
| JSN | 0.05 | 0.109 | -0.079 | 0.1 |
| KOOS Pain | **0.235** | **-0.38** | -0.1 | -0.402 |
| KOOS Sympt | 0.313 | -0.317 | -0.209 | -0.178 |
| Pain Sev | -0.12 | **0.557** | -0.086 | 0.362 |
| Pain Freq | -0.193 | **0.498** | -0.1 | 0.284 |
| Pain Imp | -0.246 | 0.281 | -0.123 | 0.091 |
| IL-1β | 0.221 | 0.274 | 0.075 | 0.023 |
| TNF-α | **-0.296** | -0.115 | -0.146 | 0.156 |
| IL-17α | 0.03 | -0.076 | -0.373 | -0.052 |
| CTX-II | 0.108 | 0 | 0.009 | -0.345 |
| Leptin | 0.055 | 0.025 | **0.573** | -0.017 |
| COMP | 0.244 | -0.012 | -0.264 | -0.417 |
| Adipo | 0.145 | 0.227 | -0.045 | -0.12 |
| PIIANP | 0.197 | 0.093 | -0.064 | -0.4 |

Spearman’s correlation analysis, with significant r values highlighted in bold (p<0.05)
