# Supplementary figures and images for: Influence of major trauma and lower limb loss on radiographic progression and incidence of knee osteoarthritis and pain: a comparative and predictive analysis from the ADVANCE study
Source: Arthritis Res Ther. 2026 Jan 26;28:49. doi: 10.1186/s13075-026-03739-4 (PMC12918490; doi:10.1186/s13075-026-03739-4)

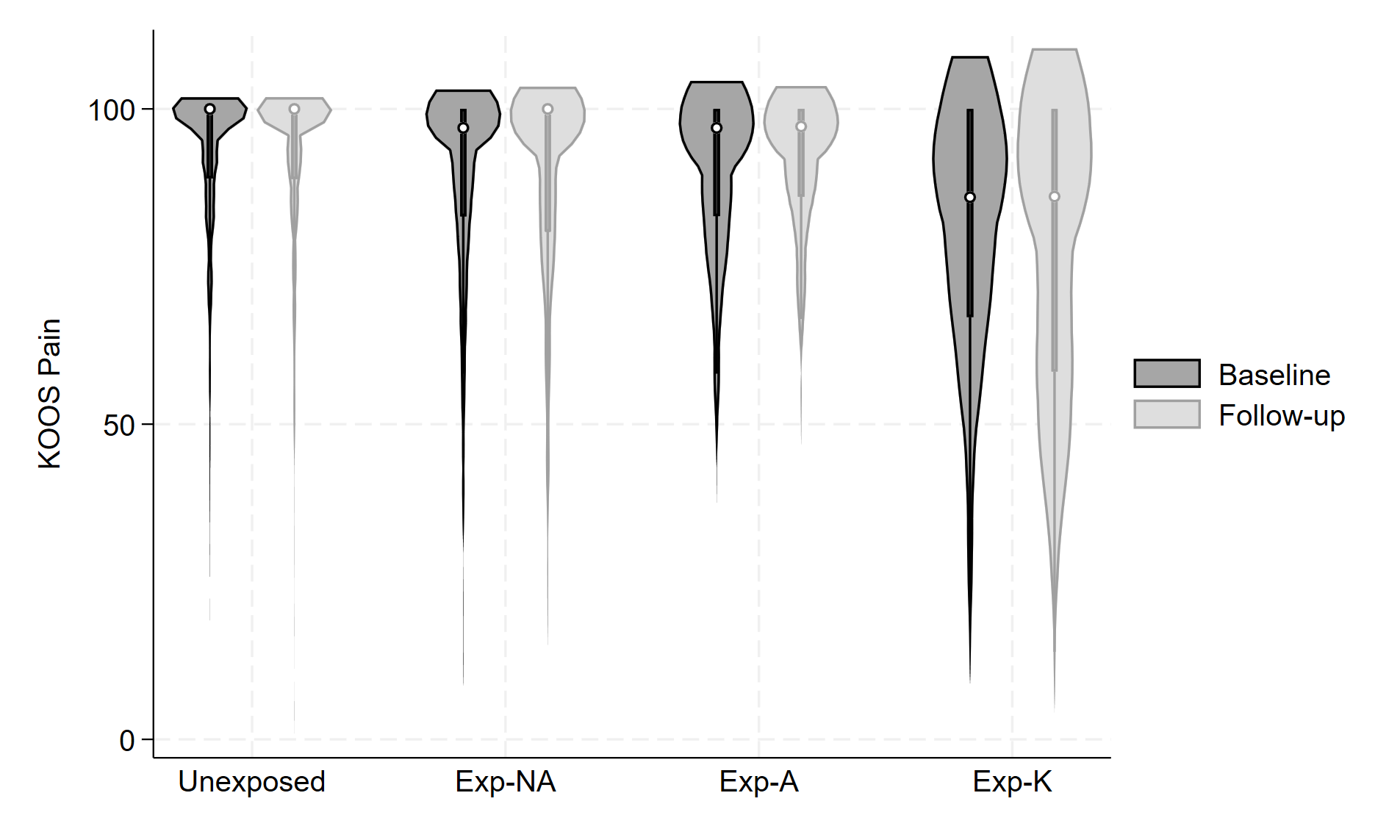

Supplement: Supplementary file 4 — Supplementary Material 4: KOOS Pain Scores. [file 13075_2026_3739_MOESM4_ESM.png]
