# Supplementary material for: Influence of major trauma and lower limb loss on radiographic progression and incidence of knee osteoarthritis and pain: a comparative and predictive analysis from the ADVANCE study
Source: Arthritis Res Ther. 2026 Jan 26;28:49. doi: 10.1186/s13075-026-03739-4 (PMC12918490; doi:10.1186/s13075-026-03739-4)
Supplement: Supplementary file 5 — Supplementary Material 5: Predictive Modelling [file 13075_2026_3739_MOESM5_ESM.docx]

Supplementary File F - Predictive modelling results

Incident rOA

Incident Pain

Progression of rOA

Progression of Pain

Pain progression for those with lower-limb loss


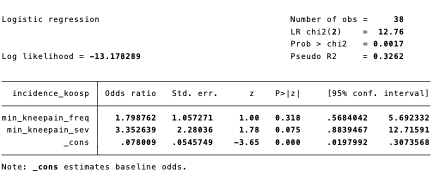


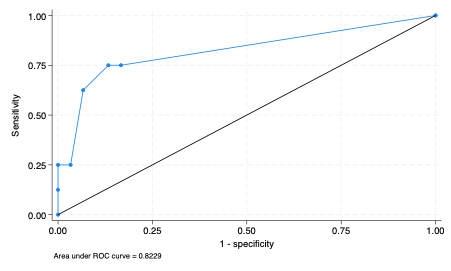


| R2 | 0.3362 |
| --- | --- |
| P | 0.0017 |
| AUROC | 0.8229 |
| SE | 0.0931 |
| AIC | 32.35658 |
| BIC | 37.26734 |
