# Supplementary material for: Influence of major trauma and lower limb loss on radiographic progression and incidence of knee osteoarthritis and pain: a comparative and predictive analysis from the ADVANCE study
Source: Arthritis Res Ther. 2026 Jan 26;28:49. doi: 10.1186/s13075-026-03739-4 (PMC12918490; doi:10.1186/s13075-026-03739-4)
Supplement: Supplementary file 6 — Supplementary Material 6: Serum Biomarker Data. [file 13075_2026_3739_MOESM6_ESM.docx]

Table 1. Baseline serum candidate osteoarthritis biomarker concentrations

|  | Total | Unexposed | Exposed | Exp-NA | Exp-A | Exp-K |
| --- | --- | --- | --- | --- | --- | --- |
|  | N=1,118 | N=553 | N=565 | N=389 | N=141 | N=35 |
| IL1β (ng/l)  Median (IQR) | 0.02 (0.02-0.06) | 0.02 (0.02-0.06) | 0.02 (0.02-0.06) | 0.02 (0.02-0.06) | 0.02 (0.02-0.06) | 0.02 (0.02-0.06) |
| TNFα (ng/l)  Mean (SD) | 1.94 (0.58) | 1.94 (0.64) | 1.93 (0.52) | 1.94 (0.56) | 1.92 (0.41) | 1.93 (0.42) |
| IL17α (ng/l)  Median (IQR) | 1.30 (0.97-1.82) | 1.27 (0.96-1.82) | 1.33 (0.98-1.82) | 1.32 (0.99-1.77) | 1.33 (0.96-1.91) | 1.23 (0.99-1.73) |
| CTXII (ug/l)  Median (IQR) | 0.20 (0.05-0.64) | 0.22 (0.05-0.67) | 0.18 (0.05-0.62) | 0.20 (0.05-0.62) | 0.15 (0.05-0.70) | 0.05 (0.05-0.37) |
| Leptin (ug/l)  Median (IQR) | 5.65 (3.04-9.29) | 5.51 (2.97-8.75) | 5.80 (3.23-9.93) | 5.68 (3.16-9.37) | 6.09 (3.16-11.67) | 8.68 (4.57-12.86) |
| COMP (ug/l)  Mean (SD) | 263.56 (88.53) | 267.07 (88.71) | 260.11 (88.30) | 280.31 (86.15) | 208.57 (76.20) | 243.25 (69.00) |
| Adipo (mg/l)  Mean (SD) | 6.33 (4.49) | 6.21 (4.01) | 6.46 (4.91) | 6.62 (5.25) | 6.29 (4.36) | 5.32 (2.33) |
| PIIANP (ug/l)  Median (IQR) | 109.10 (73.90-160.10) | 109.20 (74.60-158.30) | 109.10 (72.90-160.70) | 111.40 (74.90-168.50) | 107.10 (71.50-155.50) | 92.60 (68.10-123.40) |

IL – Interleukin, TNF – Tumour Necrosis Factor, CTX-II – C-terminal cross-linked telopeptide of type II collagen, COMP – cartilage oligomeric protein, PIIANP – N-propeptide of collagen IIA, Adipo – Adiponectin. Exp-NA: Exposed - No lower limb amputation, Exp-A: Exposed – Lower Limb Amputation, Exp-K: Exposed – Knee Injured.

Biomarker sampling, variability and quantification

At baseline, 1118 of 1145 had serum collected, 553/565 unexposed and 565/579 exposed. The 27 participants without samples were due to participant refusal, unable to obtain sample or laboratory error.

Sera underwent analysis for selected cartilage turnover biomarkers (COMP, CTX-II, N-propeptide of collagen IIA (PIIANP)), pro-inflammatory cytokines (IL-1b, IL-17a, TNF-a) and metabolic markers (leptin and adiponectin) using enzyme-linked immunosorbent assay (ELISA) or Meso Scale Discovery (MSD) by Affinity Biomarkers Lab (London, UK), accredited to ISO 15189:2012. Each plate included two kit controls and three internally identified quality control samples.

The worst reported intra- or inter-variability coefficient of variation for each biomarker was; MSD: IL-17α CV <9.5%, IL-1β <7%, TNF-α <15%; ELISA: COMP <12%, Leptin <7%, Adiponectin <8%, CTX-II <11%, PIIANP <6%.

For biomarker concentrations below the lower limit of quantification (LLOQ), a value halfway between zero and LLOQ threshold was selected, and for those above the upper LOQ (ULOQ), it was ULOQ threshold + 1. This was performed for IL-17α (<0.54=0.27, n=24), PIIANP (<5.9=2.95 n=23, >1000=1001, n=9), CTX-II (<0.1=0.05, n=421), and IL-1β (<0.043=0.0215, n=713) (20).
